# Design consideration on integration of mechanical intravascular ultrasound and electromagnetic tracking sensor for intravascular reconstruction

**DOI:** 10.1007/s11548-024-03059-5

**Published:** 2024-01-18

**Authors:** Wenran Cai, Kazuaki Hara, Naoki Tomii, Etsuko Kobayashi, Takashi Ohya, Ichiro Sakuma

**Affiliations:** 1 Graduate School of Engineering, The University of Tokyo, Tokyo, Japan; 2 Department of Oral and Maxillofacial Surgery, Yokohama City University Graduate School of Medicine, Yokohama, Japan

**Keywords:** Endovascular navigation, 3D intravascular reconstruction, Intravascular ultrasound (IVUS), Electromagnetic (EM) sensor

## Abstract

**Purpose:**

Considering vessel deformation, endovascular navigation requires intraoperative geometric information. Mechanical intravascular ultrasound (IVUS) with an electromagnetic (EM) sensor can be used to reconstruct blood vessels with thin diameter. However, the integration design should be evaluated based on the factors affecting the reconstruction error.

**Methods:**

The interference between the mechanical IVUS and EM sensor was measured in different relative positions. Two designs of the integrated catheter were evaluated by measuring the reconstruction errors using a rigid vascular phantom.

**Results:**

When the distance from the EM sensor to the field generator was 75 mm, the interference from mechanical IVUS to an EM sensor was negligible, with position and rotation errors less than 0.1 mm and 0.6°, respectively. The reconstructed vessel model for proximal IVUS transducer had a smooth surface but an inaccurate shape at large curvature of the vascular phantom. When the distance to the field generator was 175 mm, the error increased significantly.

**Conclusion:**

Placing the IVUS transducer on the proximal side of the EM sensor is superior in terms of interference reduction but inferior in terms of mechanical stability compared to a distal transducer. The distal side is preferred due to better mechanical stability during catheter manipulation at larger curvature. With this configuration, surface reconstruction errors less than 1.7 mm (with RMS 0.57 mm) were achieved when the distance to the field generator was less than 175 mm.

**Supplementary Information:**

The online version contains supplementary material available at 10.1007/s11548-024-03059-5.

## Introduction

Superselective intra-arterial chemotherapy is commonly used to treat oral cancer. A high concentration of anticancer drugs from the carotid artery needs to be delivered to the tumor-feeding artery through a catheter [1]. This process must be visualized by angiography. Recently, concerns about the overuse of ionizing radiation and nephrotoxic contrast agents have prompted the use of endovascular navigation with electromagnetic (EM) tracking sensors [2, 3]. An EM sensor can provide the position and orientation (posture) of the catheter. Integrating it with preoperative computed tomography angiography (CTA) can enable the catheter to be guided near the target [4]. However, vessel deformation can cause significant navigation errors. Although deformed vessel shapes can be detected using 3D ultrasound, blood vessels hidden under the bones cannot be detected [5]. To overcome these difficulties, in situ acquisition of intravascular information, such as the inter-branch distance and branch direction (orientation) information, after insertion using intravascular ultrasound (IVUS) may facilitate the placement of the catheter tip into the target tumor-feeding artery during catheterization. IVUS can reveal the cross-sectional shape of vessels under bones and is currently used in coronary artery disease diagnosis and percutaneous coronary intervention [6]. The integration of an IVUS catheter and EM sensor can enable 3D intravascular reconstruction allowing endovascular navigation [7, 8].

There are two types of IVUS catheters: mechanical or rotational scanning, and solid-state electronic scanning. In previous studies, a combination of solid-state IVUS with an EM sensor was used for 3D intravascular reconstruction [9, 10]. The advantage of solid-state IVUS is the reduction in vibrations during pullback; however, the catheter has a thick diameter, making its application to thin blood vessels difficult. In addition, because its axial resolution is relatively low (170 μm for 20 MHz), thin side branches may not be detected correctly. By contract, mechanical IVUS uses a thinner catheter and provides higher axial resolution (38 μm for 40 MHz), especially near-field resolution, with fewer ring-down artifacts [11]. It is suitable for thin blood vessels with diameters less than 8 mm. Therefore, using mechanical IVUS in the application of superselective intra-arterial chemotherapy is appropriate because the diameter of the artery is 3–7 mm [12, 13].

To date, only a limited number of studies have used mechanical IVUS with EM sensors for 3D intravascular reconstruction. The main engineering problem is the integration approach. The specific design must be evaluated by considering the factors affecting the reconstruction error, including interference between the IVUS and EM sensor, which has been shown to exist in the solid-state IVUS [14]. This study also evaluated the effect from the curvature of vessel and the distance from the EM sensor to the field generator for the simulation of realistic scenarios encountered during surgery.

To clarify and identify important factors to be considered in designing a system including an EM tracked catheter integrated with mechanical IVUS, we first evaluated the dependency of the interference from a mechanical IVUS on EM tracking system. We identified the required distance between an EM sensor and a mechanical IVUS transducer. Then, we compared with possible arrangements of the EM sensor and the mechanical IVUS transducer in terms of the accuracy of surface reconstruction of a rigid vascular phantom. Finally, we discussed the implications of the obtained findings for the design of an EM tracked catheter integrated with mechanical IVUS.

## Materials and methods

### Integrated IVUS catheter with EM sensor and image acquisition system

**Fig. 1:**
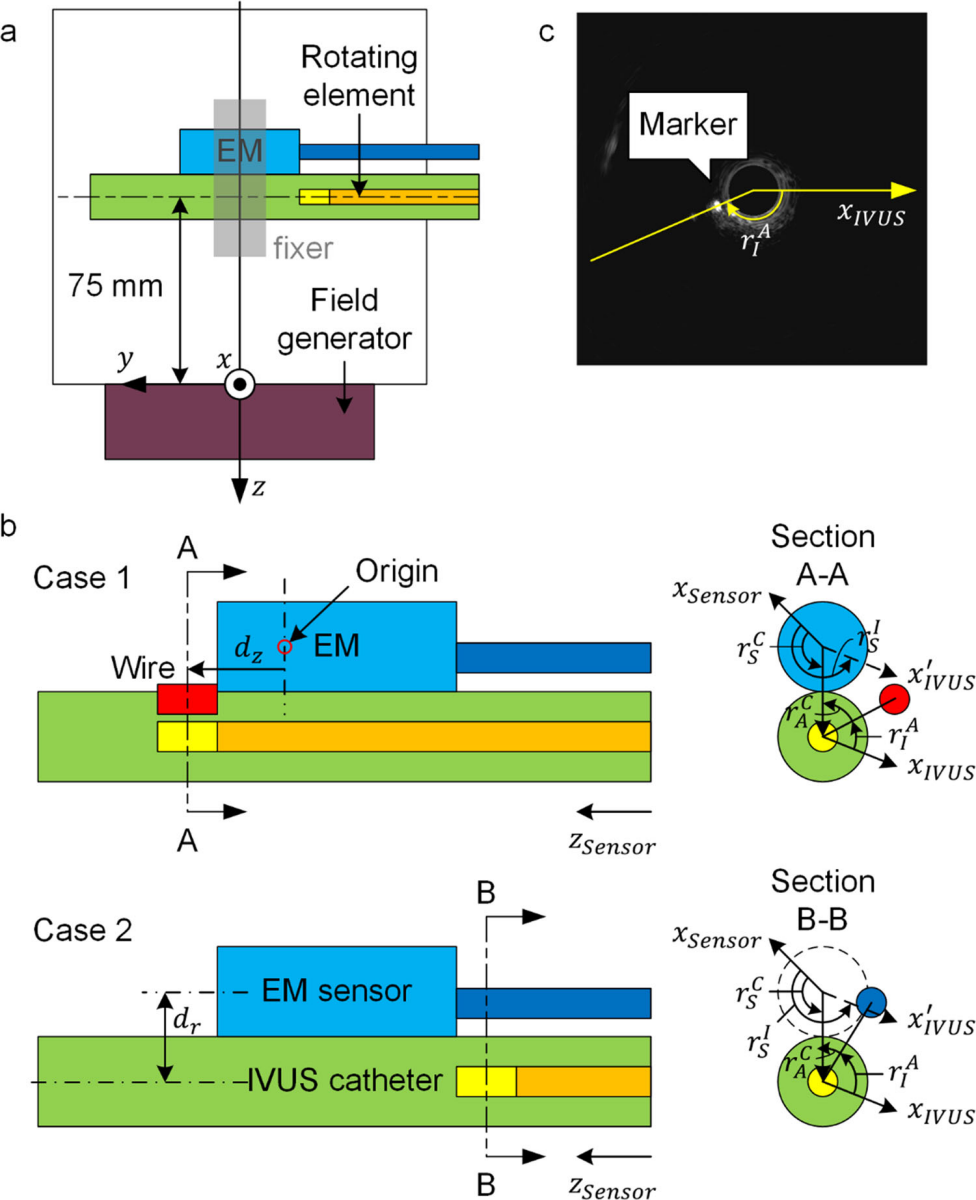
**a** Experimental setup for the evaluation of EM sensor interference caused by IVUS to the EM sensor; **b** Integrated catheter in two cases. The tape used for fixing is not shown; **c** Wire showing an artifact on an IVUS image as a dot. The contrast has been adjusted

To measure the 3D position and orientation of the IVUS catheter, a six degrees of freedom (6DOF) EM sensor (0.8 × 9 mm, NDI Corporation, Canada) was attached to the outer surface of the IVUS catheter with an EM tracking system (Aurora Tracking System, NDI Corporation, Canada) (Fig. 1a). Images were acquired using a 40 MHz IVUS catheter (Opticross, Boston Scientific, USA) with a 2.6 Fr imaging window profile. The video stream was transmitted from the IVUS system to a computer using a video capture card (DVI2USB, Epiphan Systems Inc., Canada).

### Evaluation of interference from a mechanical IVUS to EM tracking system

To quantify the amount of interference from IVUS to EM tracking system, specifically EM and mechanical (due to vibration) interference, we placed the EM sensor on a test bench along with the EM field generator of the tracking system. The distance between the EM sensor and field generator was 75 mm (Fig. 1a). The position of the field generator was adjusted such that it was on the cross section passing through the center of the catheter. The position, rotation, and indicator of magnetic field disturbances were recorded using the EM tracking system software (NDI Toolbox 5.004.015) in 10 s by changing the position of the IVUS transducer relative to the EM sensor. The indicator was defined in the software as the magnitude of interference caused by IVUS. The outputs were recorded under two different conditions:

1. Nonoperating IVUS

To evaluate the effect of the metallic components of the IVUS transducer and basal operation current of the entire IVUS system, the IVUS was not performed while the main power switch of the IVUS system was turned on.

(2) Operating IVUS

To evaluate the interference due to the driving current of the IVUS transducer unit and its rotating motion, the IVUS transducer was rotated, and image acquisition was conducted.

### Preparation of two possible arrangements of the integrated catheter

The mechanical IVUS image and EM sensor mounted on the outer IVUS catheter were not fixed in a rigid frame during pullback. The rotation of the image coordinate system relative to the outer IVUS catheter should be obtained in real time to register the IVUS images to the EM tracking coordinate system (fixed on the field generator). A fiducial marker must be integrated to determine the relative rotation. To minimize the diameter of the integrated catheter, two possible arrangements of the position for the EM sensor and IVUS transducer are proposed in Fig. 1b:

Case 1: The IVUS transducer is located on the distal side just in front of the EM sensor. An additional wire marker is integrated on the distal side.

Case 2: The IVUS transducer is located on the proximal side just behind the EM sensor. The electrical cable for power and signal transmission of the EM sensor is used as a fiducial marker.

These two possible designs were evaluated in terms of the reconstructed surface of a rigid vascular phantom. For Case 1, a silk wire was fixed approximately 0.3 mm above the IVUS transducer as the fiducial marker.

### Registration of IVUS images to the EM tracking coordinate system

The fiducial marker (silk wire for Case 1, electrical cable for Case 2) created an artifact, a dot in this case, on the IVUS image. The position of the dot was marked manually in the first frame, and could be tracked by finding the center of the area with a high intensity near it in the previous frame. The distance from the dot to the origin of the image was fixed such that the position only needed to be searched in the circumferential direction.

The origin of the IVUS transducer was regarded at the center of the black circle in the IVUS image, which could be detected using the Hough transform [7]. We connected the dot to the origin and measured the artifact angle from the x-axis to the connection line as ${r}_{I}^{A}$ (Fig. 1c). To obtain the relative rotation angle ${r}_{S}^{I}$ from the x-axis in the EM sensor coordinate system to the x-axis in the image coordinate system, the origin of the image was translated to the origin of the EM sensor (${x}_{IVUS}{\prime}$ in Fig. 1b) such that the angle ${r}_{S}^{I}$ is.

1 $${r}_{S}^{I}=\pi -({r}_{I}^{A}+{r}_{A}^{C})+{r}_{S}^{C}$$

where ${r}_{A}^{C}$ and ${r}_{S}^{C}$ are constant angles from the artifact and EM sensor to the connection of the IVUS and EM sensor counterclockwise, respectively.

As the normal vector of the image is parallel to the z-axis of the magnetic sensor, the rotation matrix of ${}_{I}{}^{S}R$ is a function of the rotation angle ${r}_{S}^{I}$ along its axis.

2 $${}_{I}{}^{S}R=\left(\begin{array}{ccc}\text{cos}{r}_{S}^{I}&\quad -\text{sin}{r}_{S}^{I}&\quad 0\\ \text{sin}{r}_{S}^{I}& \quad \text{cos}{r}_{S}^{I}& \quad 0\\ 0& 0& 1\end{array}\right)$$

Let ${}_{I}{}^{S}t$ be the displacement from the origin of the image coordinate system to the EM sensor.

3 $${}_{I}{}^{S}t={\left({d}_{r}{\text{cos}}\left({r}_{S}^{C}\right),{d}_{r}{\text{sin}}\left({r}_{S}^{C}\right),{d}_{z}\right)}^{T}$$

${}_{I}{}^{S}t$ can be obtained from the measurements of the radial distance ${d}_{r}$ and the axial distance ${d}_{z}$ between the origin of the IVUS catheter and EM sensor. These parameters can be calibrated by registration with a model of a known size (refer to the next section). Let the coordinates of one point on the lumen contour in the IVUS image coordinate system be ${c}_{I}$ and the coordinates of the corresponding point in the EM tracking coordinate system be ${c}_{W}$. The transformation from ${c}_{I}$ to ${c}_{W}$ is given by

4 $${c}_{W}={}_{S}{}^{W}R \cdot \left({}_{I}{}^{S}R\cdot {c}_{I}+{}_{I}{}^{S}t\right)+{}_{S}{}^{W}t$$

where ${}_{S}{}^{W}R$ and ${}_{S}{}^{W}t$ are the rotation and translation of the output of the 6DOF EM sensor, respectively.

### Identification of geometrical parameter

**Fig. 2:**
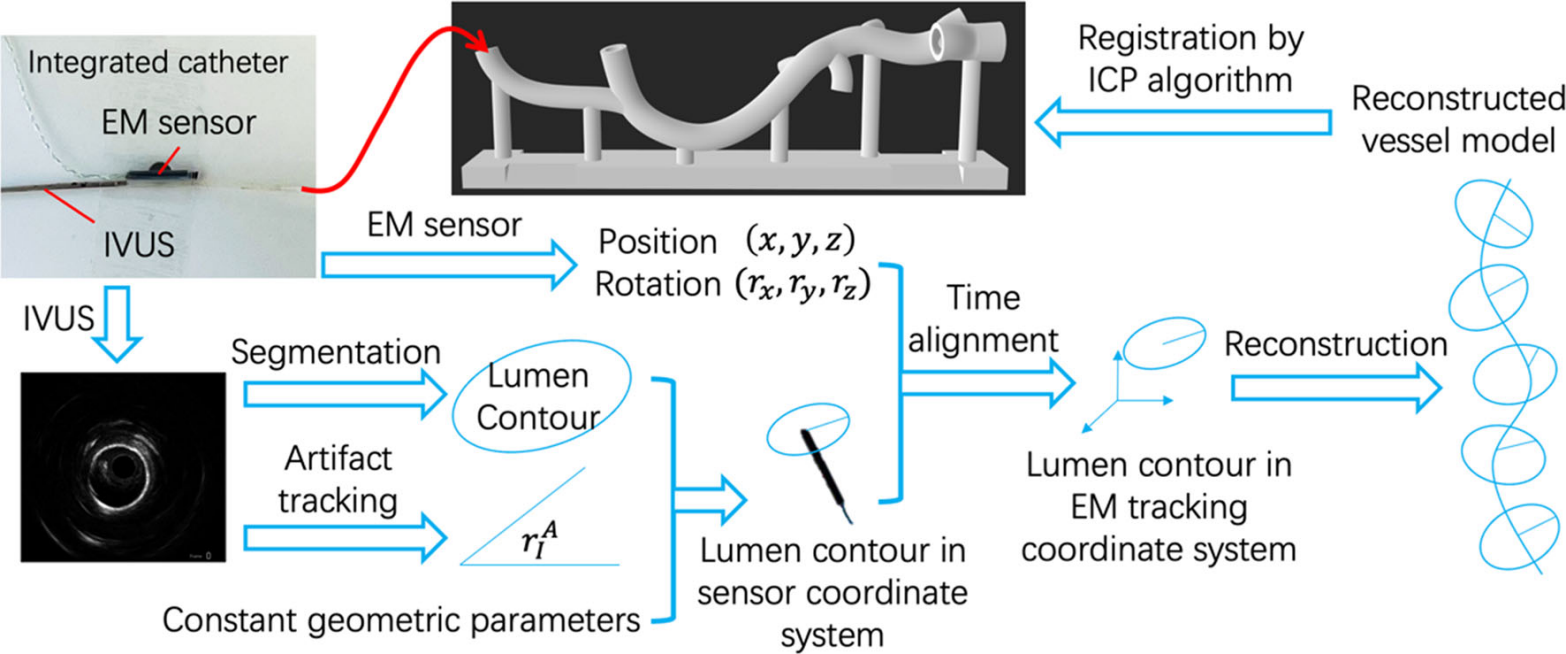
Vascular phantom and processing method for 3D intravascular reconstruction

According to Eqs. 1–4, four constant geometrical parameters, namely ${r}_{A}^{C}$, ${r}_{S}^{C}$, ${d}_{z}$, and ${d}_{r}$ should be identified for the integrated catheter to determine the transformation from the image coordinate system to the EM tracking coordinate system. ${d}_{r}$ can be determined by physical measurements. As the origin of the sensor is not located at its center and may be different for each sensor, ${d}_{z}$ cannot be directly measured. These three parameters can be calibrated using a calibration object of known size [10], which has the same shape as the vascular phantom (Fig. 2), but with all side branches removed. This process includes the following steps.

1. Estimate the range of parameters based on the geometry of the integrated catheter.
2. Pullback the integrated catheter inside the phantom (the distance to the field generator was 75 mm) with a random twist and make a reconstruction.
3. Register the reconstructed point cloud ${P}_{{\text{Reconstructed}}}$ with initial values to the dense point cloud ${P}_{{\text{Phantom}}}$ from the geometrical data of the phantom using the iterative closest point (ICP) algorithm [15] and obtain the rotation ${\varvec{R}}$ and translation $t$.
4. Calculate the mean square error (MSE) of all the vertices on the reconstruction.
  5 $$\begin{aligned}  {\text{MSE}}& =\underset{{\varvec{R}},t}{{\text{min}}}\frac{1}{2}\sum\Vert {P}_{{\text{Phantom}}}\\ & \qquad  -({\varvec{R}}\ \cdot {P}_{{\text{Reconstructed}}}({r}_{A}^{C}\text{,} {r}_{S}^{C}\text{, }{d}_{z})+t)\Vert^{2}\end{aligned} $$
5. Use an optimization algorithm to determine the minimum MSE for the optimized parameters.

### Three-dimensional surface reconstruction of a vascular phantom

A rigid phantom made of transparent resin was placed at 75, 125, and 175 mm parallel to the field generator. It was a simulated model artificially designed from a carotid artery with multiple side branches (Fig. 2). Its cross section was elliptical, with different major and minor axes in each part of the vessel (diameter range: 3–7 mm). The eccentricity was always greater than 0.8.

For each IVUS image frame, the artifact of the marker was first segmented. Subsequently, the artifact was erased from the image to segment the inner lumen contour using a method similar to the one in [7]. All the contours contained the same number of points. The distance from the center of the frame to each point should be adjusted according to the liquid and temperature [16]. The relative rotation of the wire to the image coordinate system ${r}_{I}^{A}$ was also measured from the image. Subsequently, each frame was assigned the position and orientation of the EM sensor at that moment, which enabled the calculation of the contour points in the EM tracking coordinate system.

The entire pullback process was performed manually. The pullback speed of the IVUS catheter with the EM sensor was not constant. Stick slip and reversed directional motion were observed in the experiments. By selecting the appropriate contours and connecting them, a 3D vessel model could be correctly reconstructed in real time. After each pullback, the ICP algorithm [15] was applied to register the reconstructed model with the phantom geometry, as shown in Eq. (5). The reconstruction error was evaluated by the signed distance $d$ from each vertex on the reconstructed model after registration to its closest point on the mesh surface of phantom geometry ${P}_{{\text{Closest}}}$.

6 $$d=\sigma \Vert {P}_{{\text{Closest}}}-({{\varvec{R}}}^{\boldsymbol{*}}\ \cdot {P}_{{\text{Reconstructed}}}+{t}^{*})\Vert $$

Here, when the vertex is outside the phantom geometry, $\sigma =1$; when the vertex is inside the phantom geometry, $\sigma =-1$. ${P}_{{\text{Reconstructed}}}$ is the vertex on the reconstructed model before registration. ${{\varvec{R}}}^{\boldsymbol{*}}$ and ${t}^{*}$ are the rotation matrix and translation vector, respectively, calculated by registration.

## Results

### Interference between IVUS and EM sensor

**Fig. 3:**
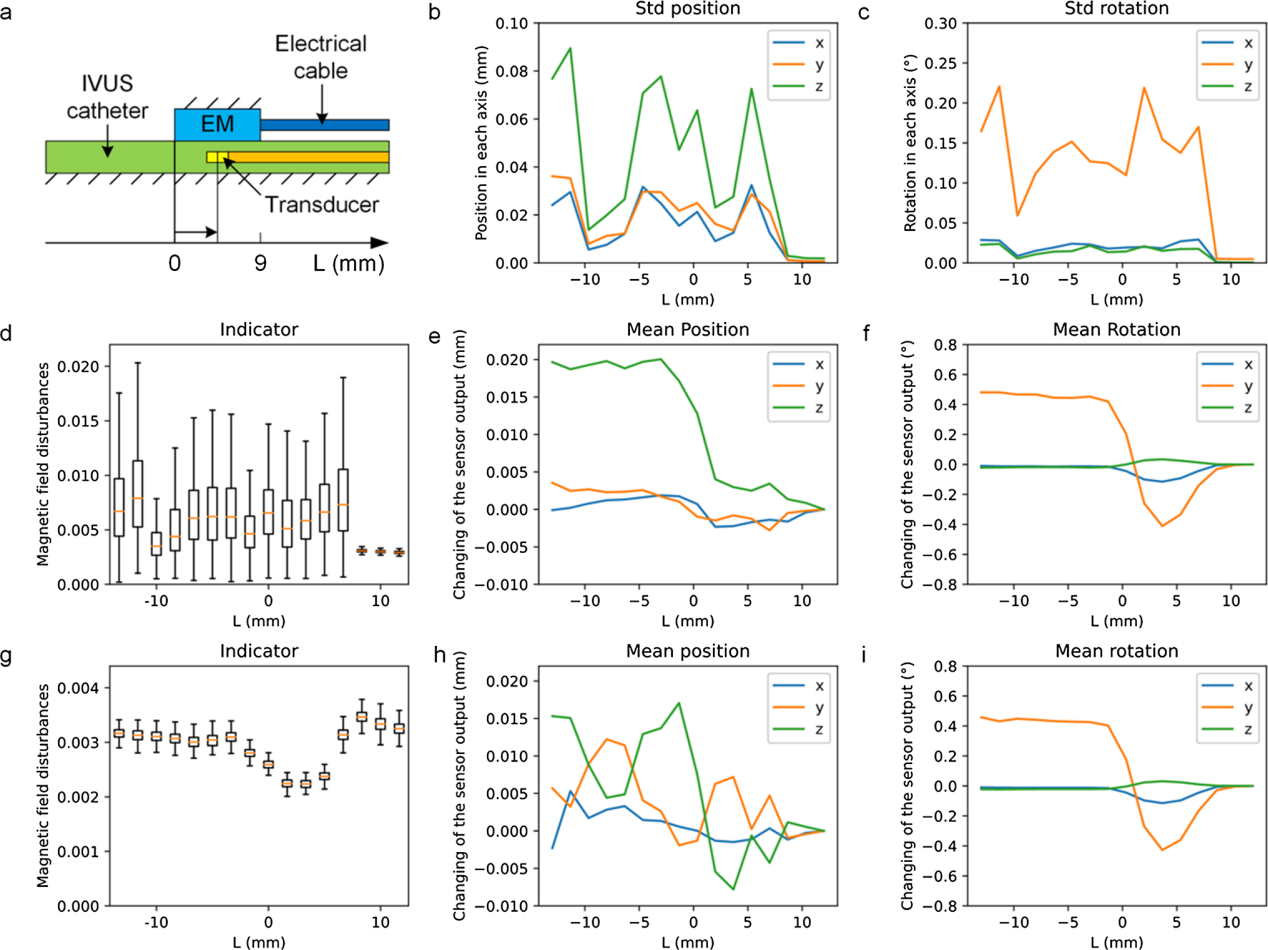
**a** Definition of the distance *L* from the center of the IVUS transducer to the top of EM sensor; **b**–**d** STDs of the position, rotation as well as an indicator of the sensor output at different distances *L* for operating IVUS; **e**–**g** Errors of the position, rotation, and indicator of the sensor output at different distances *L* for nonoperating IVUS; **h**–**i** Errors of the position and rotation of the sensor output at different distances *L* for operating IVUS

To evaluate the interference between the EM sensor and mechanical IVUS at different distances, the pullback distance *L* from the center of the IVUS transducer to the top of the EM sensor in the axis direction was varied by pulling the transducer back with the rotating element inside as shown in Fig. 3a(–13.3 ~  + 11.7 mm). *L* was measured using the motor-drive unit of an IVUS system.

The precision of the EM sensor was measured using the standard deviation (STD) for operating IVUS. The stability of the EM sensor was significantly different for different pullback distances (Fig. 3b, c). When the transducer was on the proximal side (*L* > 8 mm), the STDs of both the position and rotation tended toward zero. By contrast, the STDs were much larger when *L* < 8 mm (0.08 mm and 0.2°), and an obvious difference existed at *L* = 8 mm. In this case, the magnetic field around the EM sensor fluctuated significantly. Therefore, the output was not stable. The length of the EM sensor was 9 mm, which indicates that even when the transducer was close to the end of the EM sensor, the sensor could still provide high precision. The changes in the indicator (acceptable when less than 1) were close to the STDs obtained for the position and rotation (Fig. 3d). In this study, *L* = 11.7 mm was the reference value for the error evaluation.

After acquiring of the reference value, the position and rotation errors were measured. Even when the IVUS was nonoperating, the position and rotation of the sensor output were varied by changing the position of the IVUS transducer along the axis with a fixed EM sensor. The position in the connection direction between them and the axial rotation had the largest but acceptable error (0.02 mm and 0.5°) (Fig. 3e, f) when the transducer was on the distal side of the catheter (*L* < 0). The indicator was sufficiently small and did not show large fluctuations for the nonoperating IVUS (Fig. 3g). By contrast, the position and rotation had similar average errors but unstable position errors for operating IVUS (Fig. 3h, i). Therefore, we considered the existence of metallic components in the IVUS transducer, and the basal operation current of the entire IVUS system may cause interference to the EM sensor.

The deterioration of the IVUS images due to the EM sensor was also evaluated (data not shown). The effect of the EM sensor on the quality of IVUS images was negligible.

### Calibration of geometrical parameter

For Case 1, the STDs of ${r}_{A}^{C}$, ${r}_{S}^{C}$, and ${d}_{z}$ among five pullbacks for calibration processes were 0.4°, 0.9°, and 0.10 mm, respectively. For Case 2, the STDs were 2.4°, 2.3°, and 0.09 mm, respectively. The average values of these parameters were used to calculate the reconstruction of the vessel for error evaluation.

### Three-dimensional surface reconstruction error of a vascular phantom

**Fig. 4:**
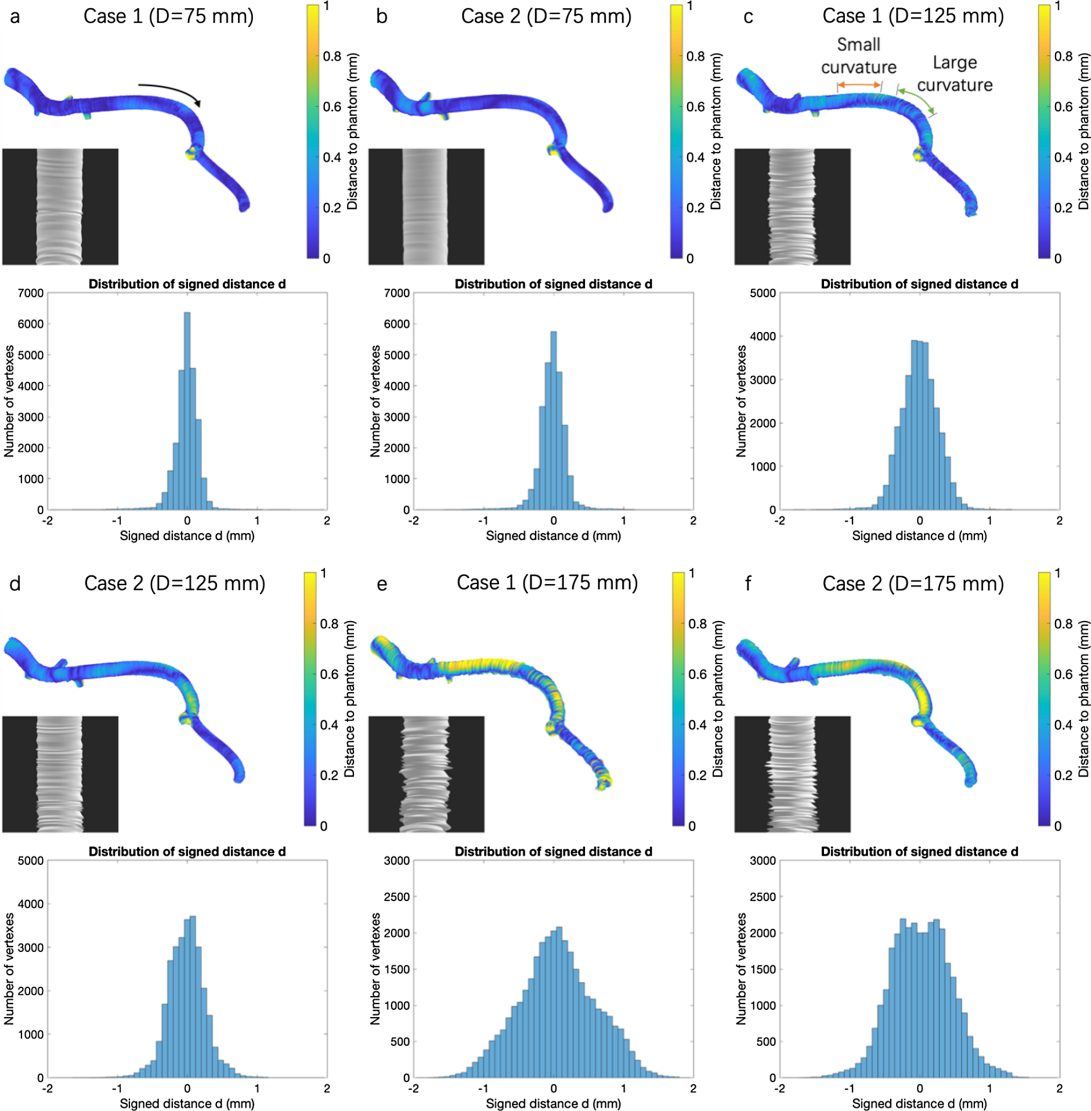
Reconstructed models and distributions of the signed distance $d$ when *D* = 75 mm (**a**, **b**), *D* = 125 mm (**c**, **d**) and *D* = 175 mm (**e**, **f**). The color shows the closest distances from the vertexes of reconstructed model to the mesh surface of phantom geometry. The distances over 1 mm in branch areas have been cut off

The integrated catheter was pulled back from left to right as indicated with curved arrow in Fig. 4a.

1. Influence of the distance between the vascular phantom and the field generator.

We reconstructed the surface of the vascular phantom both for Case 1 and Case 2 when the distance D from the vascular phantom to the field generator was changed as large as 75 mm (Fig. 4a, b), 125 mm (Fig. 4c, d) and 175 mm (Fig. 4e, f), respectively. For *D* = 75 mm, the overall surface was well reconstructed with registration errors in terms of mean absolute error (MAE) for all the vertices on the reconstruction smaller than 0.15 mm (Table 1). Case 1 had smaller error when *D* = 75 mm but larger error when *D* = 175 mm.

**Table 1:** Surface reconstruction errors evaluated based on the signed distance $d$ with different mounting positions of the EM sensor and different distances to the field generator. They are calculated from all vertexes in terms of the root mean square error (RMSE), MAE, and mean of $d$

*n* = 5	D (mm)	RMSE (mm)	MAE (mm)	Mean $d$ (mm)
Case 1	75	0.167 ± 0.007	0.117 ± 0.003	− 0.010 ± 0.001
	125	0.262 ± 0.002	0.204 ± 0.002	− 0.002 ± 0.002
	175	0.568 ± 0.022	0.453 ± 0.017	0.070 ± 0.007
Case 2	75	0.198 ± 0.008	0.136 ± 0.008	− 0.020 ± 0.002
	125	0.273 ± 0.010	0.211 ± 0.008	− 0.008 ± 0.004
	175	0.439 ± 0.010	0.354 ± 0.009	0.025 ± 0.004

2. Influence of the vascular phantom curvature on reconstruction errors.

**Fig. 5:**
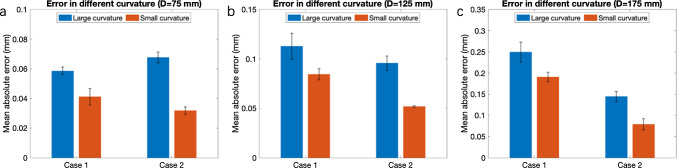
Comparison of the mean absolute error with different curvatures and distances *D* in different cases

We analyzed the surface reconstruction errors at different parts of the vascular phantom with different curvatures when *D* = 75, 125, and 175 mm. The locations are indicated in Fig. 4c as “Small curvature (radius of curvature was 30 mm)” and “Large curvature (radius of curvature was 17 mm).” Errors at locations with larger curvature were consistently larger than those with smaller curvature. The difference between errors for large and small curvature parts was more evident in Case 2 than in Case 1 for *D* = 75 mm (Fig. 5a). This tendency was also found for *D* = 125 mm (Fig. 5b). When *D* was increased to 175 mm, influence of the curvature was less evident (Fig. 5c).

**Fig. 6:**
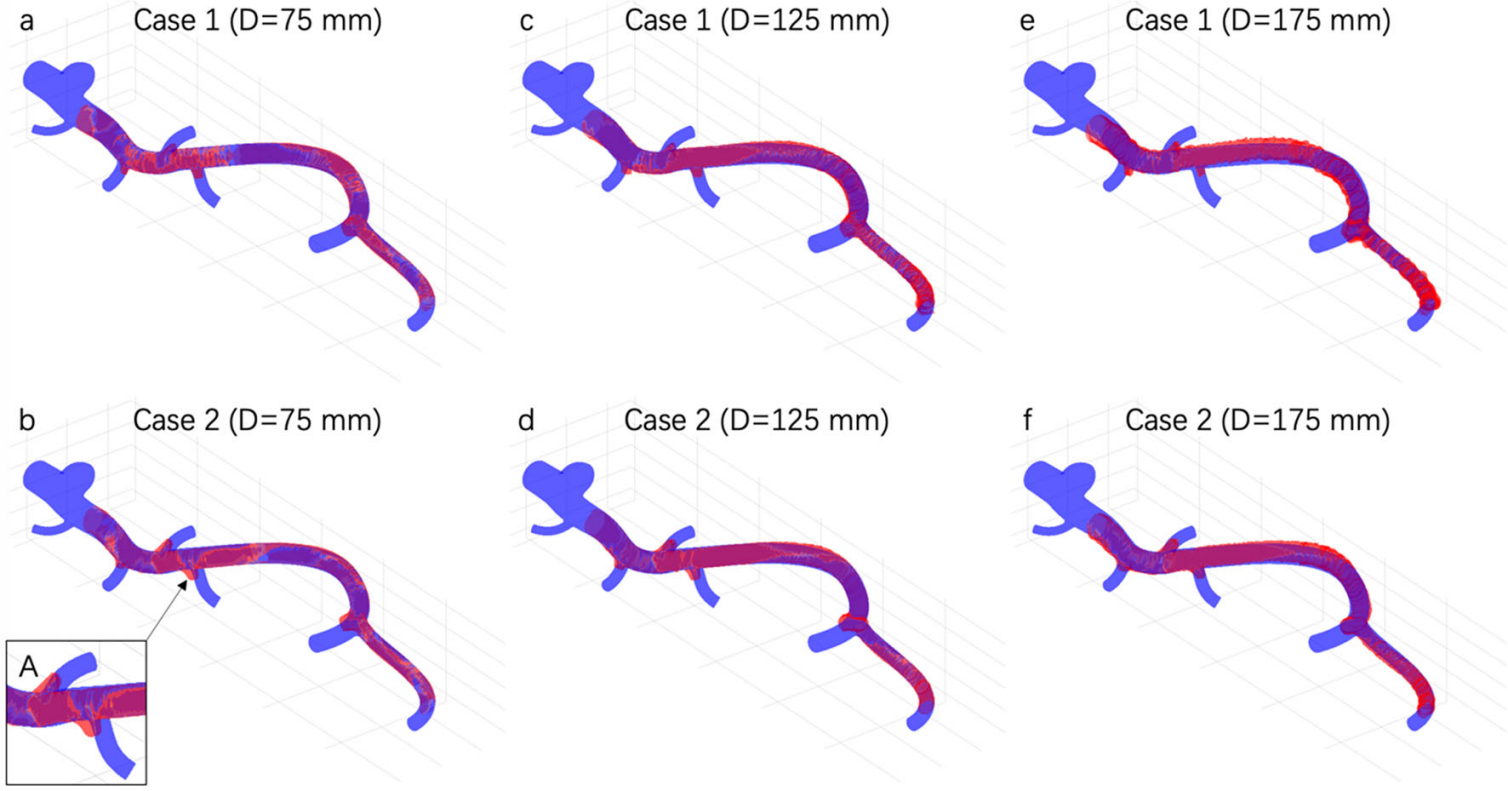
The registration results of the phantom geometry (purple) and reconstructed model (red). The enlarged area A shows the shifted side branch

When *D* = 75 and 125 mm, some side branches were obviously shifted in Case 2, with an error of nearly 1.5 mm. This shift was not obvious in Case 1 (Fig. 6a–d). However, when *D* = 175 mm, the shift was larger in Case 1 (Fig. 6e, f).

## Discussion

This study focuses on the question of how mechanical IVUS and EM sensor should be integrated in flexible catheters, using a model that approximates to reality at an acceptable level. To optimize the design of integrated catheter, three main factors have been evaluated, including experimental conditions similar to realistic scenarios. First, the magnitude of interference due to mechanical IVUS was small and not significant for designing catheter navigation. It was larger when the EM sensor was placed close to the IVUS transducer and on its distal side of IVUS catheter, which means the interference was larger in Case 1. It may be because the changes in the current inside the rotating element also affect the accuracy of EM sensor. Consequently, the reconstructed surface was smoother in Case 2.

**Fig. 7:**
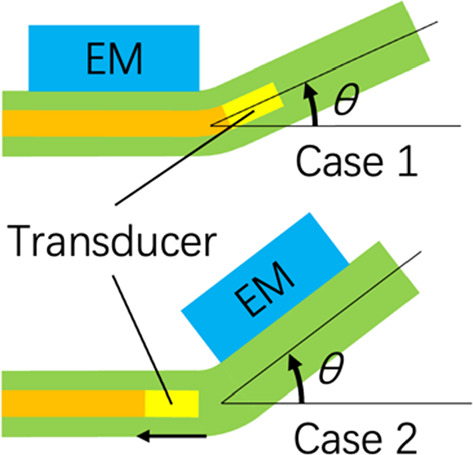
Relative motion between EM sensor and transducer. The bending angle $\theta$ of the IVUS catheter has been exaggerated

Second, in Case 2, the reconstruction error varied significantly under curvatures different from that in Case 1. Compared to the rotating element with the IVUS catheter inside as Case 1, the IVUS catheter itself is soft and much easier to bend in Case 2 (Fig. 7). Such bending invalidates the assumption that the position of the EM sensor does not change during catheter operation in the curved area. It is considered that catheter bending is the major cause of deterioration of reconstruction error and variation in the dependence of error on vessel curvature (Supplementary material 1).

In addition, we found the shift of branch location as large as 1.5 mm as shown in area A (Fig. 6b). The catheter passed through the area of larger curvature before reaching the area A. It is considered that the larger bending may cause longitudinal displacement of IVUS transducer as the black arrow shown in Fig. 7. This effect is more evident in Case 2 than in Case 1. From this point of view, Case 2 is an inappropriate design for catheter navigation application.

Third, the distance D between the vascular phantom and the field generator was also an important factor influencing the reconstruction error. The farther the sensor was from the field generator, the lower the accuracy and precision of the EM sensor. Considering the increase in the reconstruction error, the distance between the vessel and field generator should be controlled as needed, and preferably maintained less than 175 mm.

In this study, mechanical IVUS was used. It has higher acoustic frequency than that of solid-state IVUS, leading to better spatial resolution for the reason of better surface reconstruction errors than the previous study [14] (Supplementary material 2). The influence of errors in the measurement of artifact angle ${r}_{I}^{A}$ was also simply analyzed in supplementary materials 3. It is considered that this does not contribute significant reconstruction error.

The findings of the present study can be summarized as follows:

1. When the distance from the EM sensor to the field generator was 75 mm, the EM sensor interference due to mechanical IVUS was negligible, with position and rotation errors less than 0.1 mm and 0.6°, respectively.
2. The interference from IVUS transducer was smaller when the IVUS transducer was on the proximal side relative to the EM sensor (Case 2).
3. For both Cases 1 and 2, the surface reconstruction errors (MAE) of a rigid vascular phantom were approximately 0.13 mm and 0.4 mm when the distance to the field generator was set within 75 mm to 175 mm, respectively. These reconstruction errors are acceptable in application of catheter navigation for thin blood vessels.
4. Catheter bending led to changes in relative position of the EM sensor and IVUS transducer. The aspect was more evident in Case 2 and is considered to cause deterioration of reconstruction error and shift of the side branches.

Based on these findings, Case 1, in which where a mechanical IVUS transducer is placed on the distal side relative to the EM sensor, is preferred as it provides better mechanical stability during catheter manipulation in the area with larger curvature, although Case 2 is superior in terms of interference from the IVUS transducer to the EM sensor.

The experiments in this study were evaluated in an ideal situation. The navigation performance of the proposed structure should be investigated in more realistic environments, including ex vivo blood vessels. One of the disadvantages of the proposed method is that the lumen may be slightly blocked by the marker, which may cause wrong segmentation of the lumen contour and missing side branches. One solution is to register the preoperative CTA to enhance the reconstructed model including the complete lumen and all side branches. Such missing parts on the IVUS images can also be enhanced by deep learning [17]. Another limitation is the size of the EM sensor. The rigid portion of the longitudinal axis was larger than 10 mm. A method of fabricating a catheter with an integrated EM sensor and mechanical IVUS should be developed.

## Conclusion

Placing the IVUS transducer on the proximal side of the EM sensor is superior in terms of interference reduction of but inferior in terms of mechanical stability compared to the case of a distal transducer. The distal side is preferred due to better mechanical stability during catheter manipulation in the area with larger curvature. With this configuration, a surface reconstruction error less than 1.7 mm (with RMS 0.57 mm) was achieved when the distance to the field generator was less than 175 mm.

## Supplementary Information

Below is the link to the electronic supplementary material.

Supplementary file1 (PDF 144 KB)
