# Supplementary material for: Design consideration on integration of mechanical intravascular ultrasound and electromagnetic tracking sensor for intravascular reconstruction
Source: Int J Comput Assist Radiol Surg. 2024 Jan 18;19(8):1545–54. doi: 10.1007/s11548-024-03059-5 (PMC11585504; doi:10.1007/s11548-024-03059-5)
Supplement: Supplementary file 1 — Supplementary file1 (PDF 144 KB) [file 11548_2024_3059_MOESM1_ESM.pdf]

Design consideration on integration of mechanical intravascular ultrasound and electromagnetic tracking sensor for intravascular reconstruction

Wenran Cai[1, *][0000-0003-4676-4383], Kazuaki Hara[1][0000-0001-7291-6825], Naoki Tomii[1][0000-0002-7631-0940], Etsuko Kobayashi[1][0000-0003-1794-0173], Takashi Ohya[2][0000-0002-0094-2722], and Ichiro Sakuma[1][0000-0002-4919-9466]

[1] Graduate School of Engineering, The University of Tokyo, Tokyo, Japan

[2] Department of Oral and Maxillofacial Surgery, Yokohama City University Graduate School of Medicine, Yokohama, Japan

[*] Corresponding author. E-mail address: cai@bmpe.t.u-tokyo.ac.jp

S1. Detailed calculation of the error caused by catheter bending

It is considered that catheter bending is the major cause of deterioration of reconstruction error and variation in the dependance of error on vessel curvature. To prove it, we first measured the bending angle θ between IVUS transducer and EM sensor (Fig.7) and analyzed the relation of bending angle and reconstruction error. Here, the “real catheter bending” refers to the reconstruction results in the main text, and the “virtual catheter bending” refers to the reconstruction results calculated by measured bending angle. The bending of catheter was applied by a 3D printed model with constant curvature, which has the similar size as the large curvature area as Fig.4c. The inserting direction and depth for evaluation were the same as the phantom experiment. The angle was measured directly by images captured by a digital camera. The result shows that at the center point of the large curvature area, the bending angles were 7° in Case 1 and 14° in Case 2.

Then, we conducted reconstruction of virtual catheter bending based on the actual output of EM sensor pulling back inside the vascular phantom and the bending angles measured above. The trajectory of EM sensor was registered to the original mesh surface of phantom geometry. Cross-sections can be cut from the mesh surface using the points along the trajectory and the normal vector with virtual bending. The normal vector was calculated by rotating the direction of EM sensor along the binormal vector of the closest point on the centerline of phantom geometry. These cross-sections can be back projected into the EM tracking coordinate system using the direction of EM sensor without virtual bending. Finally, the reconstruction error was evaluated by the surface reconstruction errors (MAE) of signed distance d as the actual experiment of real catheter bending (Eq. 6).

To compare the result with the MAE of real catheter bending, when D = 75 mm, the reconstruction error contributed by curvature MAE_c of real catheter bending can be simply estimated as

$$MAE_c \approx \sqrt{MAE_l^2 - MAE_s^2}$$

Here, MAE_l and MAE_s are the MAEs in large and small curvature areas, respectively. By comparing it with MAEs of virtual catheter bending, it can be found they were very close in the order of magnitude (Table.S1). This result verified the hypothesis that the deterioration of reconstruction error in curvature mainly caused by catheter bending.

	Case 1	Case 2
MAE of real catheter bending	0.042 mm	0.060 mm
MAE of virtual catheter bending	0.034 mm	0.082 mm

Table.S1 Comparison of MAEs of real and virtual catheter bending.

S2. The influence of errors in different types of IVUS

In a previous study on solid-state IVUS, the reported reconstruction error was 0.64 mm [7]. In the present

study involving mechanical IVUS, the MAEs were less than 0.5 mm. When D < 125 mm, the errors were only approximately 0.2 mm. The transducer of the mechanical IVUS used in this study has an ultrasound frequency of 40 MHz, which is higher than that in solid-state IVUS (10 MHz in the previous study). Higher frequency leads to better axial resolution. It is considered to improve the accuracy. When D = 175 mm, the obtained MAEs were approximately 0.4 mm, which means that the better spatial resolution of mechanical IVUS less contributed to better reconstruction accuracy. Because of the limited penetration depth, mechanical IVUS must be used only for blood vessels with smaller diameter (3–7 mm), which is the target anatomy of intravascular interventions such as superselective intra-arterial chemotherapy. Solid-state IVUS is suitable for application in thicker blood vessels over 20 mm as shown in the previous studies.

S3. The influence of errors in the measurement of artifact angle

When performing mechanical IVUS, the rotation of the image coordinate system relative to the outer IVUS catheter should be determined. Consequently, the use of fiducial marker is essential. The measurement of artifact angle r_I^A on IVUS images can be an additional error source compared to the solid-state IVUS system. We simulated the influence of MAEs in the measurement of artifact angle specifying the location of the marker as shown in Fig.S1. It is considered that this additional error does not lead to significant reconstruction error.

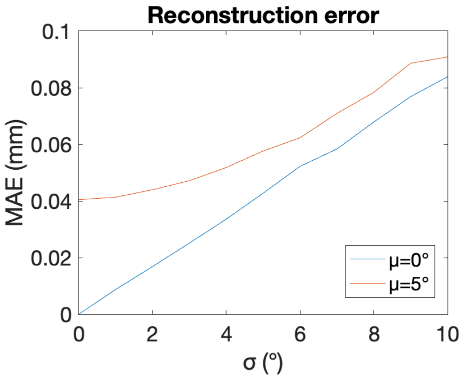

Fig.S1 Influence of noise with different means μ and STDs σ of r_I^A on average reconstruction error (n = 5).
